# Biorobotic Drug Delivery for Biomedical Applications

**DOI:** 10.3390/molecules29153663

**Published:** 2024-08-02

**Authors:** Quoc-Viet Le, Gayong Shim

**Affiliations:** 1 Faculty of Pharmacy, Ton Duc Thang University, Ho Chi Minh City 700000, Vietnam; lequocviet@tdtu.edu.vn; 2 School of Systems Biomedical Science, Soongsil University, Seoul 06978, Republic of Korea; 3 Integrative Institute of Basic Sciences, Soongsil University, Seoul 06978, Republic of Korea

**Keywords:** biorobot, biological engine, locomotion, drug-delivery systems

## Abstract

Despite extensive efforts, current drug-delivery systems face biological barriers and difficulties in bench-to-clinical use. Biomedical robotic systems have emerged as a new strategy for drug delivery because of their innovative diminutive engines. These motors enable the biorobots to move independently rather than relying on body fluids. The main components of biorobots are engines controlled by external stimuli, chemical reactions, and biological responses. Many biorobot designs are inspired by blood cells or microorganisms that possess innate swimming abilities and can incorporate living materials into their structures. This review explores the mechanisms of biorobot locomotion, achievements in robotic drug delivery, obstacles, and the perspectives of translational research.

## 1. Introduction

Nanomaterial-based delivery technologies have been extensively explored for improved therapeutic efficacy of various cargoes [1,2]. Nanomaterials can change the in vivo outcome of therapeutic cargoes and increase their delivery to pathological sites such as cancer and inflamed tissues, as well as organs such as the lungs and brain. Moreover, they can modulate the pharmacokinetic features of the cargo through controlled release at target sites. In addition to passive targeting based on physiological features, numerous studies have modified the surface of nanomaterials through specific ligands to enhance the recognition of target cells [3,4].

Despite substantial progress, nanomaterial-based drug delivery has certain limitations [5,6]. One limitation is the dependence of the distribution kinetics on the physiological features of the environment [7]. Drug-loaded nanocarriers reach targeted sites through the blood or body fluids [8,9]. Thus, the circulation rate of body fluids may govern the speed at which they reach the target site. Another limitation is poor penetration of the targeted tissues [10]. Although some fractions of the injected nanocarriers reach the target sites, the dense extracellular matrix and high interstitial fluid pressure of tissues may hinder further movement of nanocarriers inside the tissues, limiting the deep delivery of drugs within tissues [10].

**Figure 1:**
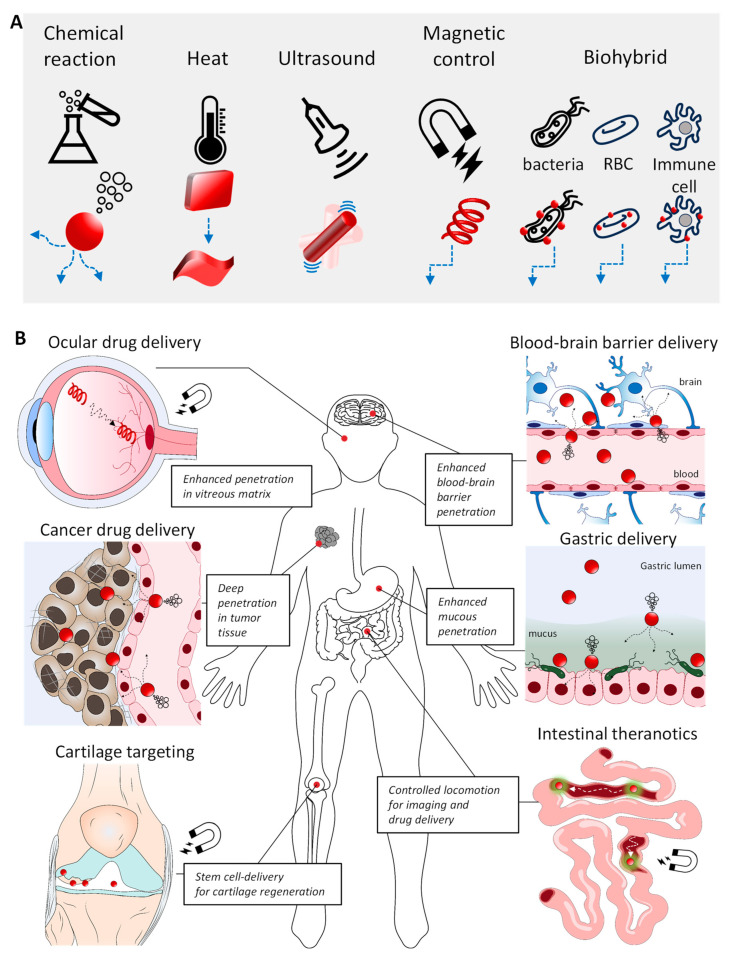
Drug-delivery systems based on biorobots. (**A**) Biorobots are designed with various shapes and engines (Red: biorobot; blue: illustrative motion path of biorobot). (**B**) Diversity of biorobots operating within various physiological environments: vitreous matrix, tumor tisssue, cartilage tissue, blood-brain barrier, gastric lumen, and intestinal lumen (Red circle: biorobot; green-glared red circle: biororobot for in vivo imaging).

Biorobotic delivery systems have recently emerged as alternatives to overcome these limitations [11]. A biorobot is a machine programmed to perform tasks automatically. Unlike long-studied nanodelivery systems without active motility, biorobots can actively move, owing to their engines. Biorobots are equipped with more than one motor engine that converts energy sources into motility [12]. Controlled movement focusing on target sites can reduce loss or undesired delivery to non-target sites. Active motion can overcome limitations of movement speed, which is governed by the physiological rate of blood circulation in the body. This review provides a summary and comprehensive perspective on the advancements and the state of the art of representative biorobotic systems since 2010. The designs of biorobotic bodies, working engines, internal cargo, and target selection are shown in Figure 1. The challenges and future of biorobots for drug delivery are also discussed.

## 2. Design of Biorobots

Biorobots must be biocompatible and biodegradable to function effectively in their target environments. The design of these robots involves the selection of appropriate materials and shapes to ensure that they can navigate and operate under physiological conditions. For example, the use of magnetic materials enables fuel-free motility inside the body, allowing robots to move without additional energy sources [13]. This magnetic propulsion can be controlled externally to perform complex maneuvers such as climbing or jumping, which are essential for overcoming physical barriers. Additionally, exploiting natural biological resources such as stomach acids can power metal-based motors, thereby enhancing the retention and function of robots within the body [14]. Further refinement of the robotic design, such as mimicking bacterial flagella with helical shapes [15], can improve the swimming performance in fluids such as physiological serum. To increase biocompatibility, surfaces can be coated with biopolymers to optimize robots for biomedical applications and ensure safe integration into biological systems. Engine components are critical for controlling the propulsion rate in the design of biorobots. As devices scale down to nano/micro sizes, controlling locomotion becomes challenging owing to the low-Reynolds-number environment and Brownian motion, which diminish the impact of inertial forces [16]. Therefore, it is essential to provide sufficient energy and ensure an efficient conversion to motion when developing such robots.

### 2.1. Materials for Biorobots

Inorganic materials, mainly nickel (Ni) [17,18] and Fe_3_O_4_ magnetic particles [19,20], are increasingly used in biorobotics because of their superior magnetic responsiveness compared to that of organic materials. These materials facilitate highly controlled and fuel-free propulsion crucial for robotic mobility. For example, Ni-coated microrobots with helical structures mimicking bacterial flagella exhibit precise swimming abilities in fluids when activated by external magnetic fields [17]. The addition of a Ti layer to these coatings minimizes their toxicity and enhances their safety for biomedical applications. One innovative use of Ni-based robots involves coating the surface with perfluorocarbon to navigate dense biopolymer structures within the vitreous of the eye, thereby improving ocular drug delivery [18]. Similarly, Fe_3_O_4_ nanoparticles have been used in nanorobots for targeted cancer therapies [19]. These robots can be remotely guided to tumors using electromagnetic fields, where they deliver chemotherapeutic agents directly to the cancer cells. Innovations include nanosized iron oxide for deeper penetration into tumors and coating robots with gold and folic acid for improved imaging and targeted therapy. Further advancements in micro-sized helical structures, combining polymers such as poly (ethylene glycol) diacrylate (PEGDA) and magnetic Fe_3_O_4_ nanoparticles, allow the precise control of drug release patterns through temperature manipulation using alternating magnetic fields [20]. This hyperthermic effect regulates drug release and enhances the anticancer properties of treatments. These developments highlight the potential of inorganic materials to revolutionize drug-delivery systems through improved design and functionality. Despite their advantages, these materials have drawbacks, such as low biodegradability and potential toxicity, requiring the development of safe post-treatment methods. Additionally, the magnetic properties of these materials can lead to stability issues, including aggregation due to magnetic interactions, which can be mitigated through surface modifications.

Biomaterials such as DNA and proteins offer distinct advantages for constructing biorobotics, owing to their biocompatibility and ability to form highly organized structures through self-assembly [21]. For example, DNA origami techniques allow the creation of complex three-dimensional structures capable of targeted drug delivery [22,23]. DNA-based structures possess several characteristics that make them ideal building blocks for constructing unique biorobots. DNA can self-assemble into complex structures through base pairing, their sequences are fully programmable, and advancements in computer simulations have allowed for the accurate prediction of their structures, which can be programmed to respond to specific molecular signals, thereby releasing therapeutic payloads in response to environmental stimuli. The development of various DNA aptamers with precise binding features enables the construction of DNA biorobotic platforms capable of specific tissue targeting and adaptable environmental sensing [24]. Protein-based robots also demonstrate significant potential, particularly in the development of motors using structural proteins such as those derived from squid ring teeth [25]. These proteins can be engineered into nanocrystals that store and release chemical fuels, enabling self-propelled movement and controlled drug release. Integrating iron oxide nanoparticles into these structures allows the movement to be directed by magnetic fields, showcasing the versatility of protein-based systems for medical applications. One advantage of protein-based structures over inorganic materials is their biodegradability, which ensures long-term safety, even though biorobots are constructed at a micro-scale. The biodegradability of protein-based materials ensures that, after fulfilling their intended function, biorobots can be safely degraded and removed by natural processes. Although biomaterials provide a sustainable and effective approach for drug delivery and actuation in biorobotics, they also face challenges related to their stability and immune responses. Enhancements in materials engineering and surface chemistry are crucial for maximizing the therapeutic efficacy and safety of inorganic and organic robotic systems.

### 2.2. Biorobot Shapes

In the challenging environment of physiological fluids, nano/micro-sized robots must be designed to efficiently convert energy into directed thrust, overcoming obstacles such as low Reynolds numbers and Brownian motion [16]. The shape of a robot is crucial for controlling its motion, and helical structures inspired by bacterial flagella are particularly effective [26]. These robots rotate in a magnetic field and are propelled forward with minimal friction. Compared to cylindrical shapes, conical shapes improve fluid maneuverability and speed, demonstrating advantages in specific biological contexts, such as the esophagus or stomach [27].

Helical robots designed with bioinspired structures are notable for their efficient swimming in high-viscosity environments such as gastric or intestinal mucus [15]. Studies have shown that Ni-based helical robots that are optimized in geometry, such as conical helices, outperform traditional microhelices in terms of speed and trajectory control under magnetic manipulation, thereby proving effective for tasks such as drug delivery and cell transport [27]. However, their relatively low surface areas limit their cargo capacity, posing challenges for their therapeutic efficacy. Additionally, complex production methods, such as two-photon polymerization, hinder large-scale manufacturing [28]. Spherical robots excel in environments where minimal contact is beneficial, such as in drug retention and release applications. They use asymmetric designs for directional propulsion, which are often powered by chemical reactions on metallic surfaces [29]. For example, magnesium-based spherical micromotors coated with pH-sensitive polymers can neutralize gastric acids and enhance drug release efficiency through the propulsion generated by hydrogen bubbles [14]. Despite these advantages, their limited interactions with biological surfaces can reduce their effectiveness in direct therapeutic applications. Rectangular robots with large surface areas are adept at interfacing with their environment, making them suitable for complex maneuvers and cargo handling. Materials such as silicone elastomers incorporated with magnetic particles enable robots to adapt to varying textures and perform diverse movements driven by magnetic fields, such as crawling or climbing [13]. These designs enable sophisticated navigation and obstacle handling, essential for targeted drug delivery in structured environments.

Each robotic shape has advantages and disadvantages that can be tailored to specific tasks within the body. Although helical robots are suitable for fluid navigation, spherical and rectangular robots are suitable for targeted delivery and adaptability, which are crucial for overcoming the physiological barriers encountered in medical applications.

### 2.3. Biorobot Engines

In the design of biorobots, the engine plays a critical role in managing the propulsion, particularly when the size of the robot is reduced to the nano or microscale level. This downsizing complicates locomotion control, owing to the low-Reynolds-number environment and Brownian motion, which diminish inertial forces [16]. Magnetic fields efficiently direct and propel robots without external fuel, making this method sustainable and nonharmful to tissues. For instance, Li et al. developed fish-like nanoswimmers propelled by magnetic fields that mimic the undulatory motion of fish to achieve high speed and precise movement control [30]. These robots can also accurately deliver therapeutic agents. However, as the robot size decreases, the propulsion power also decreases, limiting its use to micro-sizes, owing to potential capillary blockage. Moreover, although magnetic fields effectively guide robots toward targets, they lack the selectivity required to distinguish between healthy and diseased cells. Enhancements such as surface modifications can improve the targeting capabilities of robots. Magnetic thermal therapy is an effective method for inducing heat generation in internal organs by leveraging alternating current (AC) magnetic fields. Additionally, magnetic waves can create uniform magnetic fields in complex anatomical regions. Therefore, combining thermally sensitive and magnetically responsive materials in a single biorobot platform may allow the precise control of propulsion and active drug release through a thermomagnetic strategy. However, it is important to note that the use of magnetic engine-based biorobots may interfere with individuals who have implanted electronic devices such as pacemakers.

Ultrasound is another promising control mechanism, owing to its deep tissue penetration and biocompatibility. Ultrasound-driven gold nanowire motors generate movement through fluid streaming [31]. This method has been used to enhance gene silencing through rapid intracellular delivery of siRNA and for bacterial capture through acoustically propelled nanowires. Ultrasound-driven robots face limitations in areas containing air or bone, owing to poor ultrasound transmission. In addition, long-term ultrasound applications can generate unwanted heat. Therefore, time management and power optimization should be conducted before practical use.

Thermal response engines utilize materials such as N-isopropylacrylamide that undergo changes in their properties in response to temperature fluctuations [32]. These engines can release drugs at target sites where temperatures exceed the normal body temperature. However, the success of this method depends on the thermal stability of the cargo because heat-sensitive drugs are susceptible to degradation. Applications of thermally responsive engine biorobots may need to address the temperature fluctuations in the body and varying heat dissipation rates in different body parts. Temperature control must be carefully monitored to avoid thermal damage to surrounding tissues when near infrared (NIR) laser treatment is used to induce heat generation. In addition, targeting deep tissues can be challenging because of the shallow penetration of NIR lasers.

Chemical engines use reactions between metals and external chemicals such as acids or hydrogen peroxide to induce propulsion [29,33]. For example, Mg-based motors can react with gastric acid to enhance drug delivery under gastric conditions. Despite their effectiveness, by-products of chemical reactions, such as gas bubbles, may cause embolisms, restricting their use in systemic applications. Chemical engines rely primarily on the reaction rates of chemical fuels and external environmental conditions. Therefore, precise control over the release rate and number of chemical reactions over time requires the rational design of biorobot carriers. The key parameters to consider include the amount of chemical fuel, sustained reaction rates, and the formation of gas, whether directed or nondirected.

Living cells provide autonomous propulsion and can be incorporated into microrobots [34]. Bacteria such as *Magnetococcus marinus* are used for magnetotaxis and can effectively penetrate tumor tissues when combined with drug-loaded liposomes [10]. Biohybrid robots, which combine living organisms with synthetic materials, offer innovative solutions such as magnetized microalgae for fluorescence-guided therapy [35]. However, the functionality of living engines depends heavily on the environmental conditions, which can affect their therapeutic efficacy. A significant drawback of using living organisms in robotic construction is their potential for host rejection. Camouflage modifications are necessary to prevent attacks on the host immune system. In addition, ensuring consistent efficacy and performance in living engine-based biorobots can be challenging, owing to variations in batch production.

Each propulsion method has distinct advantages and limitations, necessitating careful consideration of the specific applications and environments in which a biorobot operates. Advances in micro and nanotechnology are likely to continue to enhance the efficiency and specificity of robotic systems in medical applications.

## 3. Cargo for Robotic Delivery

Therapeutic cargoes delivered by robotic devices differ in type, from small chemical compounds to biological drugs, such as nucleic acids and proteins. The loading strategies and release mechanisms of each therapeutic cargo type depend on various factors, such as the physicochemical properties of the cargoes, propulsion mechanisms of robotic devices, and targeted tissues. Many studies have developed robotic delivery systems that correspond to the delivered cargo, rational designs, and a proof-of-concept of how a drug is driven and released intentionally (Table 1).

### 3.1. Chemical Drugs

Recently, active drug release or strictly controllable release mediated by robotic delivery systems has been applied for the delivery of small chemical drugs such as doxorubicin (DOX) [33,34], 5-fluorouracil [20], and clarithromycin [29]. Small-molecule drugs are the most popular class of therapeutics used with drug-delivery systems. However, conventional drug-delivery systems for chemical drugs rely primarily on passive accumulation, which is accompanied by unexpected side effects and toxicity to normal organs. Furthermore, the immature or burst release of chemical drugs before reaching the targeted sites not only increases systemic toxicity, but also reduces therapeutic efficacy.

DOX, an anticancer drug belonging to the anthracycline group, is widely used in robotic drug-delivery systems for cancer treatment. A recent study exploited the highly porous structure of mesoporous silica nanoparticles to encapsulate DOX as a therapeutic cargo [33]. Notably, modification of silica nanoparticles with urease caused them to self-propel in the presence of a urea substrate. The generation of ammonium gas from urea catalysis by the urease nanorobot facilitated the release of the loaded DOX. The DOX content found inside cervical cancer HeLa cells was 4-fold higher than that in particles without urease.

**Figure 2:**
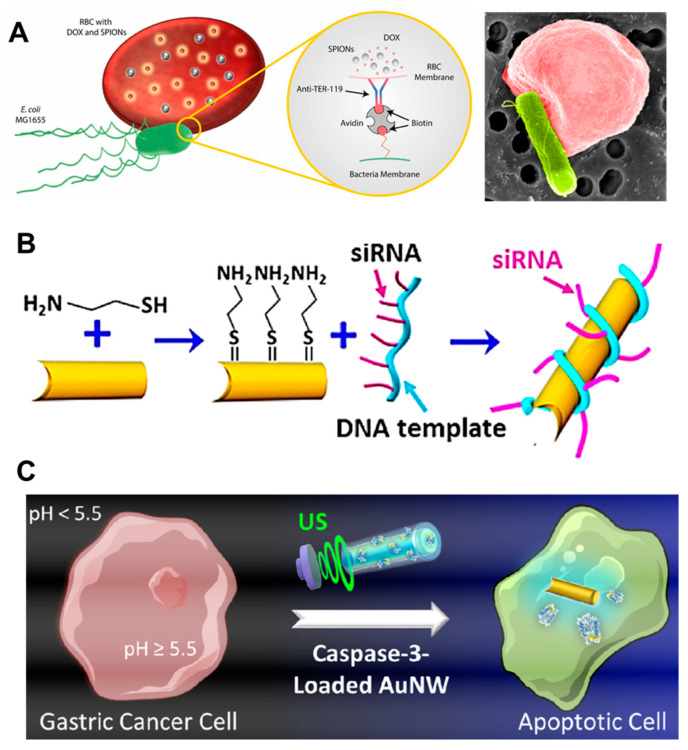
Examples of cargo drugs used in robotic delivery systems. Various payload types, including small molecules (**A**), siRNAs (**B**), and proteins (**C**) are loaded in biorobotic platforms to enhance delivery efficacy. Reprinted with permission from [30,34,39], respectively.

Another study designed an erythrocyte-based bacterial microswimmer for the delivery of DOX under low pH conditions in tumor microenvironments (Figure 2A) [34]. DOX was loaded into vesicles made of red blood cells via hypotonic treatment. Under hypotonic conditions, DOX would passively diffuse into the intracellular compartments of the erythrocytes. This drug-loading technique resulted in up to 78% DOX encapsulation efficiency without disrupting erythrocyte membranes. By attaching bacteria and iron oxide nanoparticles, the movement of the microswimmer was controlled by both magnetic guidance and actuation of the bacterial flagella. With actuation guided by bacteria, this hybrid microswimmer can penetrate small microfluidic channels that mimic the tiny capillary vessel system in the tumor environment. At this point, the low-pH environment triggers the swelling of erythrocytes, resulting in a higher release of DOX than that at physiological pH.

A floating-plan robot delivery system was designed to deliver DOX and release drugs at transiently high concentrations under NIR conditions [36]. DOX and gold nanorods were co-encapsulated in a thermally responsive hydrogel block made of N-isopropylacrylamide (NIPAM). The floating-plane skeleton was constructed from a 3D-printed polysiloxane and micropatterned cardiomyocytes. The robot movement system was assisted by the continuous contraction and restraint of a micropatterned cardiomyocyte propellant attached to the planar skeleton. Interestingly, floating-plan mobility and stationary phase can be controlled using NIR light. In the in vitro model, the floating plane placed on the surface of the cell culture medium was transiently stopped by wing transformation under NIR irradiation. This was followed by the deformation of the NIPAM gel block and the release of concentrated DOX into the cancer cells. This transiently high concentration of DOX was reported to efficiently kill cancer cells with a 5 s stop of the robot at the targeted area, while leaving the surrounding cells viable. This concept suggests the feasibility of the autonomous delivery and targeted release of DOX under controlled NIR light and cellular engines.

A biodegradable microrobot, designed for magnetically driven delivery of the anticancer drug fluorouracil (5-FU), was developed with a multimodal release system (5-FU) [20]. This microrobot incorporated both fluorouracil and iron-oxide nanoparticles into a 3D-helical structure microrobot composed of a biodegradable polymer, PEGDA, and PETA. The study utilized a rotating magnetic field to not only drive the locomotion of the microrobot but also trigger fluorouracil release in a normal, high-burst, or constant manner. Without the introduction of an alternating magnetic field, fluorouracil was released in the normal mode, which was attributed to passive diffusion of the drug from the polymer matrix. However, application of an alternating magnetic field with various parameters can produce different hyperthermia-mediated release patterns. For example, the alternative magnetic field at 430 kHz and 45 kA m^−1^ applied for 1 h induced a burst release pattern, whereas applying the magnetic field by intervals, such as for 7.5 min at 3 h, 30 min at 6 h, and 60 min at 12 h, produced a constant release pattern.

The extremely low pH in the acidic fluid of the stomach has been exploited to trigger drug release [29]. Clarithromycin, an antibiotic drug, was encapsulated in the polymer layers of the microspherical robotic particles for on-purpose delivery to kill *Helicobacter pylori* bacteria in the stomach. Robotic particles use biodegradable poly(lactic-co-glycolic acid) (PLGA) and strongly mucus-adherent chitosan as multilayer shells to protect Mg cores. Once the particle enters the acidic fluid in the stomach, the hydrogen gas generated by the reaction between the magnesium core and protons drives the self-propulsion of the particle and facilitates clarithromycin release. Using this mechanism, enhanced retention of microrobotic particles in the stomach wall was achieved, resulting in a higher antibacterial activity of clarithromycin compared with conventional microparticles without an acid-responsive engine.

Curcumin and 5-aminosalicylic acid were actively delivered to sites of gastrointestinal inflammation using a combination of two bioengineered microrobots [37]. Glucose oxidase and catalase were asymmetrically encapsulated on one side of the extracted yeast cells. *Saccharomyces cerevisiae* is the primary activator of the propulsion of this plateau. In the presence of glucose in the gastrointestinal environment, immobilized enzymes consume glucose, creating a gradient that facilitates the movement of the microrobot through the mucus barrier of the intestinal wall. Following the transportation of microfold-cell transcytosis, they are delivered to inflammatory sites by being engulfed by macrophages and subsequently delivered to inflammatory sites through the chemotactic property of the cells. Through this mechanism, the accumulation of curcumin and 5-aminosalicylic acid increases up to 1000-fold in the inflamed stomach and colon tissues.

In addition to conventional chemical drugs, the targeted delivery of radionuclides using nanorobots for therapeutic and imaging purposes has been investigated [38]. Iodine-131 (^131^I) was loaded onto mesoporous silica nanoparticles powered by a urease engine. The generation of an ammonium ion gradient and CO_2_-based motility have the potential for drug delivery to urea-rich tumor environments, such as bladder cancer. The intravesical injection of ^131^I-nanorobots resulted in positive bladder tumor suppression, even at low doses. This self-propelling system addresses the challenges encountered in conventional treatments, in which therapeutic outcomes are limited, owing to poor drug retention in the bladder.

Most studies have demonstrated promising anticancer effects using in vitro models. However, in clinical practice, the tumor tissue is covered by a large number of adjacent cells. Nanosized propellers are likely to be advantageous for tissue penetration and intracellular drug delivery. In contrast, a micro-sized robotic system may face a challenge in solid tumor accumulation because it cannot take advantage of the enhanced retention and permeation effects in tumor tissues, which is a strong advantage of nanosized delivery systems. To overcome this issue, a combination of controllable micropropellers that can trigger the release of the drug/carrying nanorobots at the peripheral site of the tumor tissue, allowing further penetration, is a promising candidate for clinical applications.

### 3.2. Genes and Nucleic Acids

Gene therapy involves using nucleic acids to treat inherited disorders and cancers. Gene therapy requires intracellular delivery of gene materials to patient cells, which is challenging because of the negative charge of nucleic acids. Various delivery systems have been developed to enhance the delivery of DNA and RNA therapeutics using physical methods, such as electrophoresis, or gene carriers, such as nano/microparticles. However, these delivery systems have limitations in terms of transfection efficacy and nonspecific delivery, which may lead to unexpected side effects. A highly controlled delivery system, that not only improves transfection efficacy but also selectively delivers gene material for human use, is desired. Previous studies have reported robotic delivery systems for gene materials that exploit highly controllable stimuli to direct gene carriers to target cells or areas [30,42]. Robotic carrier propulsion can be strictly controlled or navigated to target sites using external stimuli such as a magnetic field or ultrasound. Moreover, the stimuli facilitated the interaction between the robotic carrier and the targeted cell, thus improving internalization.

Qiu et al. developed a magnetic-field-powered microswimmer to deliver plasmid DNA [42]. Inspired by the natural motility of bacteria, the microswimmer functions as an artificial bacterial flagellum that steers, moves, and interacts with targeted cells through the wireless control of a low-strength rotating magnetic field. The microswimmer body, with a dimension of 5 × 16 μm, was fabricated with 3D-laser printed polymer and coated with metallic Ni/Ti bilayer. A complex of the DNA plasmid, Lipofectamine, and cell-adhesive proteins was absorbed onto the surface of the microswimmers. With 3D navigation using a low-strength rotating magnetic field, transfection can be precisely controlled under a microscope. With micrometer-scale precision, the authors directed the movement of the microswimmer toward the target and transfected the cells without affecting the adjacent cells. In addition to in vitro applications, the application of this transfection system in human diseases, where the target tissue is located in a hard-to-reach area, holds promise. However, to actualize this potential, addressing concerns regarding the removal of particles from the human body is imperative, given that the microswimmer’s body is made of nonbiodegradable materials.

In another study, siRNA transfection was enhanced by actuating an ultrasound-powered nanomotor (Figure 2B) [30]. Rolling circle amplification was used to generate a DNA scaffold and siRNA was hung along the DNA scaffold using an antisense sequence. Similar to other gene delivery methods, siRNA delivery also faces challenges such as cell penetration and nonspecific transfection. In this study, gold nanowires were used to carry an siRNA/DNA scaffold and deliver it to cells through acoustic movement under ultrasound irradiation. The gene silencing efficiency in cells treated with the nanomotor and subsequently subjected to 5 min of ultrasound treatment was 4.3-fold greater than that observed with static carrier treatment. Although ultrasound application and electroporation share the same final purpose of enhanced siRNA internalization, ultrasound treatment is more advantageous because it causes less damage to cell membranes. Compared with conventional siRNA transfection agents such as Lipofectamine, this method demonstrates superior efficiency. The ultrasound-assisted propulsion of the nanomotor induces fast carrier internalization. This results in gene knockdown within 24 h as opposed to the 72-h incubation required for transfection with Lipofectamine [49].

Biorobots also have potential applications in CRISPR/Cas9-based gene editing. The delivery of Cas9 protein complexed with a single guided RNA (sgRNA) requires intracellular internalization, followed by endo/lysosomal escape. In a recent study, an ultrasound-driven nanomotor was designed using gold nanowires to deliver a Cas9/sgRNA complex [44]. With the assistance of ultrasound, the self-propelled nanomotor resulted in a 95% targeted gene knockout within 24 h of treatment, surpassing the 60% gene knockout achieved with conventional lipofectamine carrier treatment for 48 h. Although the experiment was conducted on the B16F10 cell model, this study provides a potential approach for Cas9-based gene editing in animals for future studies.

The delivery of messenger RNA (mRNA) using light-driven nanomotors has recently been demonstrated [43]. This system was designed to overcome intracellular barriers encountered in therapeutic nucleic acid delivery, including the endolysosomal compartment. An azobenzene-derived lipid was introduced into lipid-based nanoparticles to facilitate light-responsive molecular movement. They undergo molecular switching between cis- and trans-isomer forms under UV–Vis light exposure and thus act as mechanical pendants for the movement of nanoparticles. Upon exposure to light, the mechanical motility of the azobenzene pendant groups causes particle movement and disrupts the endolysosomal membrane, freeing it of mRNA cargo. A 2.6-fold increase in mRNA transfection was achieved with azo-lipid nanoparticles with light assistance compared to that observed with Lipofectamine. Notably, the azolipid nanoparticles exhibited negligible cellular toxicity for in vivo transfection.

Although robotic devices assisted by external stimuli have many advantages for delivering gene materials, some concerns must be addressed before translating these methods for human use. First, most studies performed transfection in vitro, in which cells were plated in culture dishes. Hence, the movement of these robotic devices in two dimensions could be easily controlled. Transfection of in vivo tissues is more challenging because the targeted cells are usually packed with adjacent cells or tissues. The narrow space between the cells may limit the full actuation of microsized motors. Furthermore, ultrasound-assisted biorobots only work efficiently in local tissues, which require penetration enhancement; however, the challenge of delivering these biorobots to targeted tissues remains unresolved. One of the proposed solutions to this challenge is the combination of ultrasound-responsive nanorobots embedded in targeted carriers, such as bacteria [50]. In some studies, ultrasound has been used as the main power source to drive the actuation of robotic devices. Long-term exposure to ultrasound induces DNA breakage and damage [51,52]. For ultrasound applications in humans, the optimal time for inducing gene silencing using gold nanomotors must be carefully considered.

### 3.3. Therapeutic Proteins

Various robotic delivery systems have been designed to enhance protein therapeutic efficacy. Therapeutic proteins are difficult to deliver because of their rapid degradation and high molecular weights. However, their poor stability during degradation or cleavage by extracellular or intracellular proteases limits their efficacy. Moreover, in cases where the targets of therapeutic proteins are located inside the cells, intracellular delivery is inefficient.

Intracellular delivery of caspase 3 enzyme by an ultrasound-assisted nanomotor to induce apoptosis in gastric cancer cells has been reported (Figure 2C) [39]. Caspase 3 is a promising therapeutic protein that cleaves and activates key cellular proteins that play critical roles in programmed cell death or apoptosis [53]. The intracellular delivery of caspase 3 faces challenges due to the negative charge of protein molecules, their heterotetrameric states, and the vulnerable nature of their active sites [54]. In a study by Avilia, ultrasound-powered gold nanowire carrying caspase 3 in a pH-responsive matrix polymer-coated shell facilitated the intracellular delivery of this enzyme into cancer cells. It is known that the extracellular pH of gastric tumors is slightly acidic (pH 5.5–6.7) [55,56], whereas the intracellular cytoplasmic pH is neutral (pH~7.5) [57]. The pH-responsive polymer EUDRAGIT L30 D-55 was exploited for the protection of caspase 3 owing to the insolubility of this polymer at acidic pH. Once the nanomotor is internalized into the cell, the dissolution of the polymer shell at neutral pH releases caspase 3 into the cytoplasm and induces cancer cell apoptosis. These studies reported that up to 80% apoptosis was observed in nanomotor-treated AGS cells that were subsequently subjected to ultrasound treatment, surpassing 24.4% cell death observed in non-ultrasound-treated cells. This study suggests a promising nanomotor platform for the intracellular delivery of not only caspase 3 but also other therapeutic proteins.

In a study by Wei and colleagues, Staphylococcal α-toxin antigen was delivered as an oral vaccine by utilizing a biomimetic self-propulsion micromotor [40]. Oral vaccines have attracted great interest, owing to their convenient administration and ability to elicit a broad immune response mediated by mucosal immunity [58]. However, the major limitation of oral delivery of protein vaccines is the short retention time of the vaccine in the gastric intestinal tract, which reduces the stimulation efficacy of vaccines against mucosal immune cells. In this study, the Staphylococcal α-toxin, an antigen derived from *Staphylococcus aureus*, was entrapped in biocompatible red blood cell membrane to mask the toxicity of the antigen. In order to equip the system with an engine, the antigen/cell membrane vesicles were co-encapsulated with Mg/TiO_2_ core under the cover of chitosan and Eudragit L100−55. This design allowed the vaccine microparticles to pass through the acidic fluid of the stomach. Once the vaccine motor reaches the low tract of the intestine, the dissolution of Eudragit layer at neutral pH would facilitate water penetration to activate the Mg/TiO_2_ motor. The vaccine micromotor was strongly attached to the apical side of the intestinal wall, assisted by a chitosan layer. This study reported that the antitoxin IgA titer was one order of magnitude higher in mice treated with vaccine microparticles than in those treated with static vaccine microparticles.

**Figure 3:**
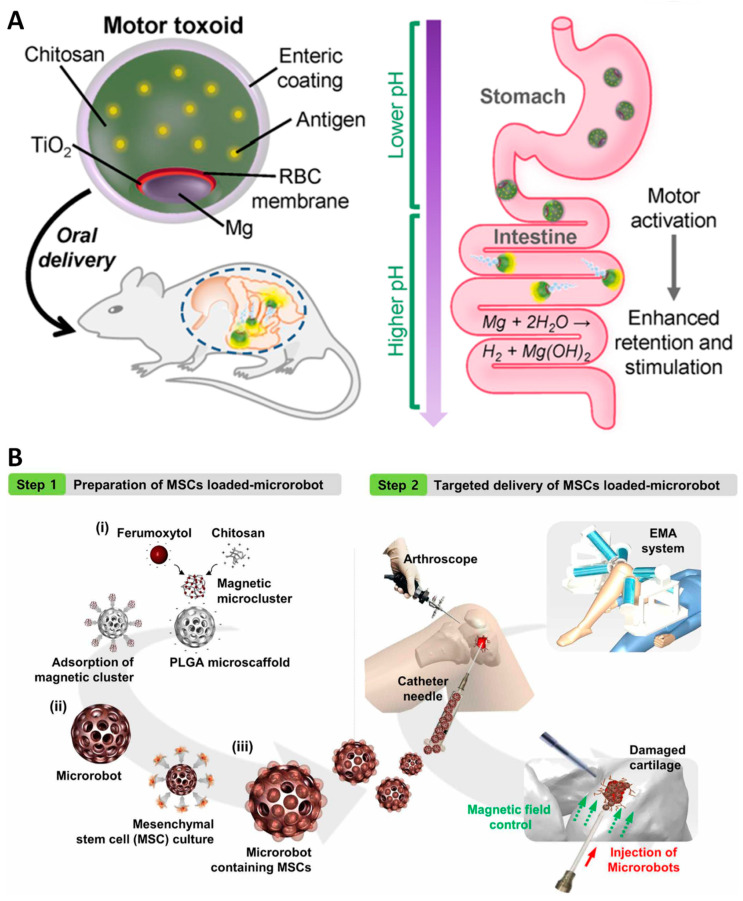
Design of biorobotic platforms for protein and cell delivery. (**A**). An oral vaccine based on an autonomous microrobot for delivery of Staphylococcal α-toxin antigen [40]. (**B**) Construction of a microrobot using chitosan-PLGA scaffold for delivery of mesenchymal stem cells into damaged cartilage with assistance of an electromagnetic articulography system (EMA). PLGA-based microrobot was prepared by absorption of feromoxytol-chitosan microcluters (i) following the loading of mesenchymal stem cells (ii). (iii)The microrobot was injected into the knee joint, and its movement was controlled by an EMA system [46]. Reprinted with permission from [40,46].

A thrombin delivery system using nanorobots made of DNA origami has been reported [24]. Thrombin is a key coagulant that induces intratumoral vascular thrombosis. In this study, self-assembled DNA origami featuring nucleolin-targeting aptamers was designed as a thrombin delivery platform. The thrombin was attached to the inner face of the DNA origami sheet using an antisense polyadenosine linker. Notably, in the inactivated form, the rolling of the DNA sheets protects thrombin from its activity. Upon interaction with nucleolin, a protein highly expressed in tumor epithelial cells, the DNA sheet is triggered to unfold, exposing thrombin, initiating coagulation within the tumor tissue, and ultimately inducing tumor necrosis (Figure 3A). A DNA nanorobotic system with targeting and triggering release properties may inspire the future design of novel therapeutics modified with different ligands.

Macrophage-mediated and magnetically controlled biorobots have recently been developed for cytokine delivery [41]. Poly L-lysine-coated iron oxide nanoparticles loaded with IL-12, CCL-5, and CXCL-10, which are cytokines required for T-cell differentiation and monocyte recruitment, were attached to the surface of the macrophages. The resulting hybrid robot exhibited both the chemotactic motility of immune cells and magnetically propelled movements. The concentration of iron-oxide nanoparticles was optimized to avoid toxicity to transporter cells, and in vitro magnetic control demonstrated that the hybrid robot could follow the assigned paths, circumventing the obstacles posed by cancer cells in an in vitro setup. However, the release of loaded cytokines, their practical movement within living tissues, and their interactions with T cells and other immune cell components require further investigation.

### 3.4. Cells

Stem cell therapy is a promising therapy with applications in various diseases such as tissue damage, tissue degeneration, and inherent diseases [59,60]. Effective stem cell therapy requires the successful homing of transplanted cells to targeted tissues, where the cells can survive, differentiate, and proliferate properly to execute the desired functions. The direct injection of cells into the target tissue may be the simplest and most convenient method. However, the difficulty in precisely guiding the needle to the targeted area is a challenge, in addition to the low cell viability of this method [61]. Surgical implantation is another option for stem cell deposition; however, this method may be limited to easily exposed tissues or non-risky areas, where surgery does not place a large burden on patients. A stem cell delivery system that can bypass the tissue barrier is wirelessly controllable, while maintaining good cell viability. Recently, several studies have developed robotic devices scaled down to the micro-scale, which are designed to transport stem cells to targeted sites.

Recently, a microrobot with a 3D structure reassembling from a 3D scaffold was constructed to deliver MC3T3-E1 fibroblasts and stem cells [45]. The authors used 3D-laser lithography printing to generate a burr-like porous spherical structure. This design was developed to minimize the ratio of the surface area to viscous resistance during flow in the bloodstream, indicating a higher magnetic response during body use. The porous structure of this scaffold, which mimics the extracellular matrix, maximizes the cell loading capacity, nutrient supply for tissue vascularization, and cell growth. Notably, because the backbone of the microrobot was coated with a metallic Ni layer, the movement of the cell-carrying microrobot was precisely demonstrated in a zebrafish embryo model without affecting host survival. Using HeLa cells, they observed successful release of the transplanted cells. This concept provides a promising 3D autonomous platform that mimics the tissue matrix for stem cell delivery. To demonstrate its feasibility for human use, it is necessary to perform guided deposition of cells in more complicated models, such as mouse and rabbit models. Successful delivery of stem cells with proper functions in these models is a novel strategy for stem cell therapy.

To overcome the biocompatibility and safety challenges of metallic micro-sized delivery robots, a recent study developed a biodegradable actuator made of a PLGA scaffold as a controllable vehicle for the targeted delivery of human adipose-derived mesenchymal stem cells (Figure 3B) [46]. To equip a porous scaffold with magnetically responsive features, the authors embedded a complex of feruxytol and chitosan into a PLGA matrix through static interactions. Feruxytol is a dextran-coated iron-oxide nanoparticle approved by the FDA for human use as an imaging contrast agent [61]. Human adipose-derived stem cells can grow in scaffolds to form cell spheroids and proliferate in vitro for up to 16 days without signs of toxicity. More importantly, this study established an electromagnetic articulography (EMA) system equipped with microrobot injection tools and an arthroscope to monitor the in vivo targeting procedure. In a rabbit knee-cartilage regeneration model, microrobot-carrying stem cells were injected into the knee at the site of injury. The 3D movement control of the microrobot in the knee using the EMA system was monitored by manipulating the microrobot to firmly adhere to the defective area of the injured cartilage, where the stem cells grew and executed their tissue recovery functions. In this concept, biodegradable and clinically approved materials were used to construct microrobots. Together with the evidence of stem cell delivery in animal models, this system has potential applications in clinical trials involving cartilage regeneration.

Cells are typically transported by microrobots, with the cells adhering to the surface of the delivery system. A recent study developed a capsule-type microrobot to encapsulate delivered cells in a carrier compartment [47]. This design allows the cells to be protected from attack by immune cells as well as from cell detachment caused by the fluid shear force during movement. Interestingly, the microrobot was shaped into a helical structure and comprised two parts: a plunge and a cap. The “pick and drop” functions were controlled with magnetic field, which involved “picking” the cell from donor place and transferring it by “dropping” at another place. This innovative approach has curbed the exploration of novel concepts for cell encapsulation and transfer into culture dishes. An extended experiment conducted using in vivo models would be useful for fully evaluating the potential applications of this system. For example, a microrobot system can be used for the in situ transfer of healthy stem cells to damaged tissues for regeneration. This study demonstrates the feasibility of controlling a single microrobot to approach and capture targeted cells for delivery. It becomes imperative to optimize the simultaneous “pick and drop” control of this system in broader applications requiring simultaneous delivery of multiple cells.

Chimeric antigen receptor (CAR) T-cells have also been used for delivery via magnetic acoustic actuation [48]. By adopting metabolic labelling with azide-tagged carbohydrate moieties, CAR-T cells were engineered with magnetic beads, yielding a hybrid microrobot version of CAR-T cells (M-CAR-Ts) with deep penetration into the tumor tissue under a programmed magnetic field. This concept addresses the current limitations of CAR-T cells in solid tumor treatment, in which physical barriers hinder CAR-T cell infiltration. In addition, magnetic beads can be engineered with costimulatory proteins to boost M-CAR-T cell activation. CD19-SPCA1 tumor-bearing mice intravenously received M-CAR-T cells following magnetic recruitment to the tumor tissue for up to 12 h. Local acoustic treatment allowed deeper penetration of M-CAR Ts by up to 6.6-fold, compared with the non-actuated version. The robotic CAR-T cell concept is a potential treatment strategy for other solid tumor types.

## 4. Target Diseases

### 4.1. Cancer

Cancer is a significant burden for patients because few effective therapies are currently available to achieve a complete cure. Several studies have shown that a shift in the redox balance and disordered redox signaling are key factors contributing to malignant progression and therapeutic resistance. Tumor cells persistently produce large quantities of reactive oxygen species (ROS) as a consequence of alterations in oncogenic factors, such as genetics, metabolism, and the tumor microenvironment. An increase in ROS levels in tumor tissues has been linked to factors associated with cancer progression, such as DNA alterations, cell proliferation, apoptosis resistance, and metastasis [62,63]. Recent studies have shown that elevated ROS levels in tumor tissues can be tolerated by modulating the antioxidant pathways. In addition to redox homeostasis, tumorigenesis is promoted by the tumor microenvironment, which involves both cellular and non-cellular factors. Targeting the tumor microenvironment is an attractive strategy in recent cancer treatment approaches [64,65]. Owing to the common characteristics of redox dysregulation in tumor tissues, the expression of various unique factors, such as the extracellular matrix, excess metabolites, and marker proteins, has been utilized as a target for the design of robotic delivery systems.

Biorobotics have been developed by exploiting oxidative stress in tumor microenvironments. These robotic delivery systems are not only designed to take advantage of hydrogen peroxide catalysis for their movement but also to neutralize the oxidative stress conditions in tumor tissues.

**Figure 4:**
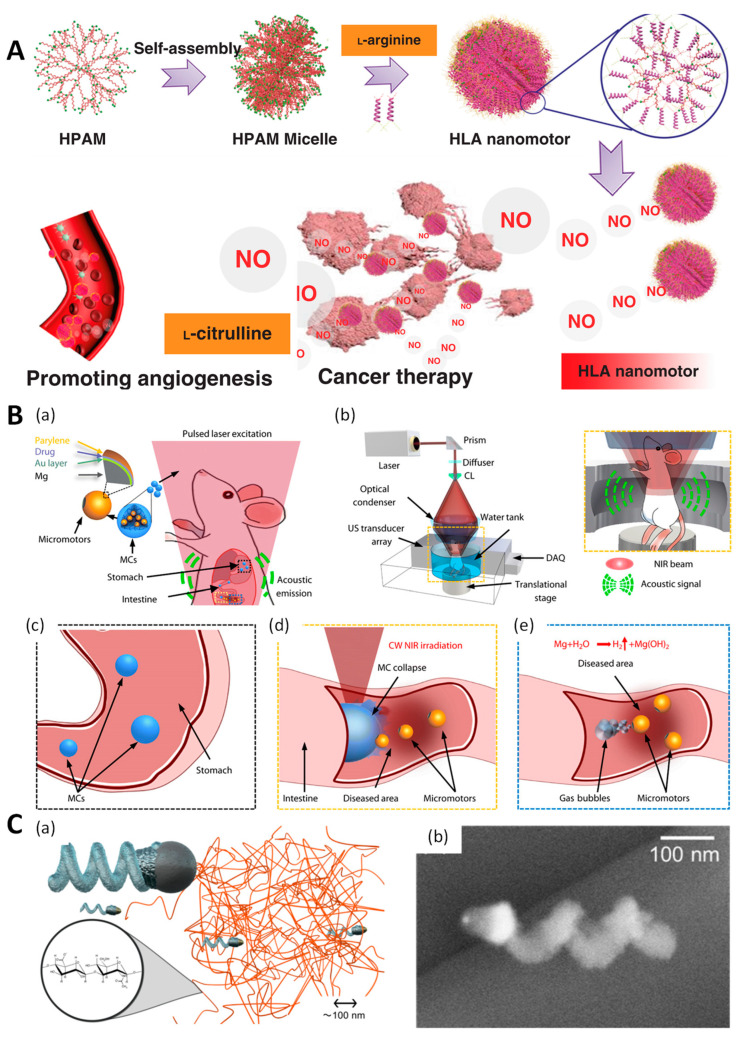
Representative robotic devices allowing active delivery of therapeutic drugs or cells to the targeted sites in various disease treatments. (**A**) NO generation by L-arginine in the tumor microenvironment propels the nanoparticles and induces anticancer effects. Reprinted with permission from [70]. (**B**) Navigation of micro-propeller by photoacoustic computed tomography for targeted delivery in gastrointestinal diseases (**a**). A set up of photo ascoutic computed tomography for gastric intestinal imaging (**b**). Micro-propeller is protected in gastric environment by enteric coating (**c**) and activated in intestine lumen by NIR irradiation (**d**). Exposure of Mg layer induced gas generation driving biorobot’s motion (**e**). Reprinted with permission from [71]. (**C**) The helical nanopropeller is able to penetrate the high-viscosity matrix, which is crucial for drug delivery in vitreous environment. (**a**) Illustrative motion of helical propeller and (**b**) helical shape was imaged by transmission electron microscopy. Reprinted with permission from [72].

Oxidative stress-derived metabolites in the tumor microenvironment have been targeted for chemical reactive engineering [66,67,68]. For example, the chemical catalysis of hydrogen peroxide using a Pt layer generates a burst release of oxygen, which functions as a propellant [68]. Catalase-assisted biological catalysis has been used to decompose hydrogen peroxide. In these studies, catalase was immobilized on a particle surface using a Janus design [66] or encapsulated in a hollow core of bowl-shaped particles [67,69]. Oxidative stress in tumor tissues results in the production of large quantities of hydrogen peroxide, which is considered one of the hallmarks of oxidative stress and is exploited as a fuel to trigger the propulsion of microrobots. Reactive oxygen species, which are ubiquitous products of oxidative stress in tumor tissues, in addition to hydrogen peroxide, have been employed for the catalysis of NO generated from L-arginine [70]. A nanomotor backbone was built using poly-L-arginine as the fuel for continuous NO production (Figure 4A). Gas generated from these engines propels carriers into tumor tissues and facilitates drug release from the scaffold [33].

Current chemotherapy for female reproductive cancers such as ovarian or cervical cancer lacks specificity and is associated with numerous side effects [73]. Targeted drug delivery to the female reproductive tract has gained increasing attention because of its use in sperm-based biohybrid robots. In a recent study, biohybrids of sperm and 3D printed materials were used to magnetically guide anticancer drug delivery [74]. Considering the application of sperm-based micromotors for cancer treatment in the female reproductive tract, sperm cells are outstanding candidates compared with synthetic micromaterials because they naturally mimic swimming in such environments. Sperm membranes protect drugs from immature release, immune attacks, and enzyme degradation. In a study by Xu et al., magnetic guidance controlled the movement and uncapping of a 3D-printed magnetic tubular microstructure, thus releasing sperm when it hit the tumor cell walls. The fusion of cancer cells and sperm cell membranes is a unique feature of this system as it facilitates the intracellular delivery of anticancer drugs. However, several limitations must be addressed. For example, controlling sperm cell survival, fertilization risk, and compatibility of sperm origin should be carefully considered in clinical practice.

Some studies have attempted to modulate the tumor microenvironment using extracellular proteases to trigger drug release using biorobotics. Matrix metalloproteinases (MMPs) are extracellular proteases found in tumor tissues that play critical roles in extracellular matrix remodeling, angiogenesis, and tumor progression. MMP-2 is secreted by tumor cells to degrade collagen, the main component of the extracellular matrix, facilitating escape from the primary tumor site and promoting metastasis. Therefore, elevated MMP-2 levels in tumor tissues have been used to activate drug release in many studies [75,76]. A helical microrobotic swimmer composed of collagen was developed to exploit the high levels of MMP-2 in tumor tissues [77]. Using magnetic guidance, the helical swimmer carried model therapeutic and diagnostic cargo to approach the tumor tissue. Under MMP-2 enzymatic activity, the swelling and degradation of collagen by MMP-2 resulted in the release of macromolecular drugs and imaging agents. This mechanism facilitates the detection and suppression of tumors.

### 4.2. Gastrointestinal Disease

Patients with gastric diseases such as *Helicobacter pylori* infection, gastric ulceration, and enteritis receive prominent therapeutic treatment with ingestible drugs. The drug-delivery systems approved for these treatments are primarily ingestible tablets, capsules, or oral solutions. However, these oral treatments are limited by the passive diffusion, low absorption, and short retention of drug molecules in the gastrointestinal tract. The gastrointestinal tract is dynamic and transports food and drugs via peristaltic movements. Therefore, one of the greatest challenges in gastrointestinal disease treatment is the lack of proper residence time for therapeutics in the target areas.

*Helicobacter pylori* is a common infection affecting half the world’s population. This results in chronic stomach inflammation that may eventually lead to peptic ulceration and gastric cancer [78]. Current guidelines suggest the use of a combination of antibiotics and proton pump inhibitors to reduce gastric acid levels for the treatment of *H. pylori* infection. Oral administration of antibiotics through passive delivery systems makes it difficult to eradicate *H. pylori* for several reasons, such as poor stability of antibiotics in gastric acid, low concentration of antibiotics in the mucus layer where *H. pylori* is located, insufficient exposure time of the bacteria, and antibiotic peristaltic movement.

*H. pylori* infection was effectively eliminated by the oral delivery of a clarithromycin-assisted gastric-acid-sensitive autonomous propeller [29]. The antibiotic clarithromycin was protected from the gastric mucosa using a Eudragit polymer. The acid-sensible micromotor was self-propelled and firmly attached to the mucus layer by equipping the microparticles with a magnesium hydrogen-gas-generation engine. Hydrogen gas generation also facilitated antibiotic release and improved the local concentration of antibiotics, effectively eradicating *H. pylori* in the stomachs of infected mice. Moreover, rapid neutralization of the acidic environment by proton depletion eliminates the need for co-treatment with proton pump inhibitors. This concept holds promise as an active drug-delivery strategy for the treatment of *H. pylori* infections. Further studies are required to compare the antibacterial potency of this strategy with that of standard therapies to elucidate its efficacy and safety for long-term use in humans.

Deep tissue imaging in gastrointestinal diseases is one of the challenges associated with the development of a reliable system for the real-time visualization and control of therapeutics. A recent study demonstrated the feasibility of using a photoacoustic computed tomography (PACT)-guided microrobotic system to track locomotion in the mouse gut (Figure 4B) [71]. The drug-loaded microrobot was administered orally, and its migration was monitored in real time. PACT imaging has a spatiotemporal resolution, deep penetration, and high anatomical contrast, allowing the exact tracking of microrobot migration in the gastrointestinal tract. Once the particle reached the targeted site, near-infrared light irradiation deformed the particles and activated the magnesium engine in the intestinal fluid to drive mucus layer insertion for prolonged microrobot retention times, with subsequent drug release (Figure 4B).

The treatment of gastrointestinal diseases has reached a new era, and the development of autonomous microrobots with self-propelling engines has been reported. Their mobility and navigation have been validated as safe in animals because chemical reactions and particle insertion into the gut walls do not affect the anatomical structures of the intestinal walls. Nevertheless, because most oral drug deliveries require multiple administrations in the short or long term depending on the disease, the safety of repeated dosages of this system needs to be guaranteed for further use in humans.

### 4.3. Ocular Disease

In ophthalmology, ocular drug delivery plays an important role in the treatment of diseases, such as diabetic retinopathy, diabetic macular edema, macular degeneration, and glaucoma. Conventional drug-delivery systems, such as topical applications, are popular for the treatment of the anterior eye. Systemic and intravitreal injections in the posterior part of the eye have poor efficacy and low drug accumulation because of the lacrimal fluid–eye and retina–blood barriers. Passive diffusion of therapeutics into the retina is slow and the retina is sensitive to degradation. Recent studies have investigated the development of microrobots for effective drug delivery via intravitreal administration.

The high-viscosity barrier of intravitreal environments has been overcome using nanosized nanorobots powered by a magnetic field [72]. The size of the robotic system is a critical factor that affects the movement and delivery efficacy of the vitreous matrix. A previous study revealed that the vitreous matrix has a mesh size of approximately 550 nm [79]. Thus, a robotic system with dimensions smaller than 550 nm can easily move and navigate through the vitreous matrix with minimal hindrance. A helical nanopropeller with a diameter of 120 nm and length of 400 nm was constructed from silica particles coated with Ni for a magnetic response. In a highly vicious medium, the nanopropeller outperformed large helice control in terms of step-out frequency. Therefore, the navigation and movement speeds of this carrier are expected to be suitable for intravitreal delivery. However, the small size of the nanomotor may limit its drug-loading capacity.

In another study, the treatment of retinal disease via active drug delivery was achieved using an appropriate design or a magnetic propeller at the micro-scale (Figure 4C) [18]. Increasing the propeller size is beneficial because the actuation and drug-loading capacities are limited to nanoscale platforms. Therefore, increasing the size and maintaining a good penetration of biorobotics into highly viscous and porous biological fluids is challenging. Similar to previous studies and inspired by its helical shape, the micropropeller had a spherical head with a diameter of 500 nm to match the pore size of the vitreous matrix. However, microparticles may face a significant challenge owing to the interaction between the particle surface and matrix collagen fibrils. This study proposes a coating method to make a micropropeller with a perfluorocarbon surface slippery in a vicious environment. This coating material is widely used in medical applications for surface modification to ensure the biocompatibility of devices [80]. In the porcine eye model, the micropropeller traveled 15 mm from the center of the eye to the retina in 30 min. Compared to other studies using passive particles, the 340-nm albumin/hyaluronic acid particles required 6 h for passive diffusion in the 10 mm range [81].

These studies suggest the active concept of wireless, controllable delivery systems for ocular diseases. Although proof-of-concept studies have been performed to address the biophysical barriers of intravitreal systems, it is necessary to examine their drug-loading capacities and release. Most importantly, the current materials used for these robotic devices are biocompatible, but not biodegradable. The fate of these devices in the ocular space may raise major concerns in terms of long-term post-therapy safety, because the large material clearance in this area is highly restricted.

### 4.4. Brain Disease

Drug delivery to the central nervous system (CNS) is challenging. The brain and other parts of the CNS, such as the spinal cord, are protected by the blood–brain barrier (BBB), which is a complex network of blood vasculature. The BBB strictly controls the diffusion and exchange of metabolites in the CNS. Therefore, it is difficult to deliver high concentrations of drug molecules to the brain. Not all macromolecular therapeutics such as recombinant proteins, peptides, antibodies, DNA, and RNA can passively diffuse into the brain. The most common misconception is that small molecules can penetrate the BBB. Indeed, more than 98% of small molecules fail to cross the BBB [82]. Therefore, approaches to improve drug delivery to the CNS are required. Various strategies have utilized rationally designed nanodelivery systems to facilitate BBB penetration. For example, cell membrane-based nanoparticles [83,84], lipoprotein nanoparticles [85], exosomes [86], and virus-based delivery systems [87] have been developed. These strategies mostly rely on biomimetic carriers that specifically target the BBB receptors. Pathological leakage of the BBB occurs in some diseases such as brain tumors, Alzheimer’s disease, Parkinson’s disease, opening of tight junctions, inflammation, and endothelial cell function collapse [88,89,90]. This BBB leakage may facilitate the passive diffusion of nanoparticles. However, pathological leakage occurs at certain stages of the disease; thus, the delivery of these nanoparticles may not always be effective. Biorobotics have been developed for efficient drug delivery through the BBB.

In a recent study, active drug delivery through the BBB was achieved by exploiting the high glucose consumption in the brain. A promising treatment for brain diseases can be realized using the glucose-gradient-sensitive nanoself-propeller developed by Joseph et al. [91]. Glucose is the most effective substrate for crossing the BBB because the brain consumes 20% of the glucose in the body [92]. The polymersome was utilized to carry a dual-enzyme-based engine, triggering propulsion of the polymer under glucose gradient conditions. Glucose oxidase and catalase are coentrapped in polymersomes. Glucose oxidase catalyzes the conversion of glucose to D-glucono-d-lactone and hydrogen peroxide. Hydrogen peroxide is subsequently converted to water and oxygen by catalase. Owing to the asymmetric topology of polymersomes, the escape of oxygen from one side of the particles drives active diffusion through the BBB. This study revealed high accumulation of polymersomes across the BBB in a rat model, which was four-fold higher than that of non-enzyme-equipped polymersomes.

Although the concept demonstrated the feasibility of enhanced delivery with a glucose gradient at the BBB, there are concerns regarding the immature activation of this system in the bloodstream, where the glucose level is always maintained at 4–7.8 mM. In addition, because the nanopropeller consumes glucose as fuel for its engine, glucose deprivation during treatment is apparent, which may lead to potential side effects on brain function. This effect may be transitional; however, repeated dosing in the treatment of brain diseases should be carefully considered.

## 5. Robotic Drug Delivery System in the Context of Internet of Things

The Internet of Things (IoT) refers to the systemic connectivity and intercommunication of electrical devices, featuring the unique identification of each device. The IoT has applications across various sectors, including consumer goods, industrial operations, and healthcare services [93]. In medical applications, the IoT is still growing, but it is promising for future healthcare systems. The internet of medical things (IoMT) includes medical devices and applications interconnected within a cloud platform healthcare system. The IoMT aims to improve the diagnosis, analysis, and remote monitoring of caregivers while empowering patients through wireless connectivity, particularly in home healthcare scenarios, offering automation and personalized medicine. The evolution of the IoMT has also encompassed advancements in drug-delivery systems. Pioneering studies have explored the integration of drug delivery with the IoT. For example, a 3D-printing technique was utilized to personalize drug dosages based on patient information within a short time, regardless of the location of the patient [94]. In a recent study, an IoT-based drug-delivery system detected the onset of epilepsy and provided an in-time injection of therapeutic drugs at a patient-condition-dependent dose [95]. These studies demonstrated that the application of the IoMT in robotic drug-delivery systems would provide several benefits in terms of improving treatment efficacy and economic aspects for both patients and healthcare providers.

The conceptualization of a robotic delivery system is primarily envisaged for the treatment of chronic diseases such as cancer, diabetes, cardiovascular disease, and rheumatoid arthritis. The treatment of these diseases involves long-term persuasion and multiple dosage intervals. The IoMT provides continuous information transfer from patients to healthcare using cloud data. With the support of real-time monitoring and analysis of patient conditions, administration time and precise dosages can be prescribed to patients from a distance. Furthermore, remote control through an internet connection allows physicians to operate the actuation or movement of the robotic delivery system into the target tissues from a distance. For instance, various robotic delivery systems that control external stimuli, such as rotated magnetic fields or ultrasound, have been developed [10,46,96]. Real-time control of these stimuli from a distance is mediated by device connections and wireless data transfer. The activities of the robots were carefully monitored through data transmitters and receivers, as well as online analyzers. Notably, patient imaging during treatment or administration can be achieved using in vivo imaging techniques, such as near-infrared imaging, ultrasonography, X-rays, or photoacoustic imaging [97,98].

Second, the drug release pattern of the corresponding robotic drug-delivery system was wirelessly controlled depending on the purpose of treatment. Remote control of the release pattern of fluorouracil in cancer treatment by adjusting the magnetic field parameters, such as frequency and interval, is useful for automated dosing [20]. For example, in patients who should receive a drug with a burst release for a quick response or sustained release for long-term effects, stimulation of the robotic DDS system for the corresponding release patterns could be programmed to match each personnel’s treatment plan.

Third, the therapeutic response of a single patient to each condition was recorded and stored in a cloud system, where big data were collected from a large population of patients worldwide. Accessible data can be shared between institutions and analyzed to provide suggestions for more effective treatment. Based on these data, artificial intelligence and deep-learning systems can be used to propose an optimal treatment protocol for each robotic drug-delivery system [99,100]. Taken together, the integration of the IoT and robotic delivery systems will result in a win-win situation for both healthcare providers and patients. Patients can receive personalized treatment with maximum comfort at home. Meanwhile, the IoT increases multitasking efficiency, thus saving time and workload for health workers.

Despite the numerous advantages of the IoT in robotic drug-delivery systems, many concerns still need to be addressed. The IoT requires strong and stable communication between action units (nano/microrobotics), corresponding sensors (health parameter sensors, drug concentration sensors), and external devices (stimuli generators and signal transmitters). Such an integration of multiple components results in unexpected treatment outcomes if a single component has a failure or error. An alarm system with an on-time stoppable action function in this autonomous system prevents unexpected situations from affecting the patient’s health.

## 6. Challenges and Future Perspectives

As stated previously, micro- and nanomotor-equipped biorobots continue to exhibit promising results. Robots that enter the body to treat diseases, which are often seen in movies, are becoming a reality. There are several concerns to consider when designing and using biorobots.

External stimuli have been studied in various ways as fuel materials in motor engines. The special environment of the body can also be utilized as fuel for engines; however, this is limited to a few organs. Currently, the most commonly used magnetic field is evaluated as a possible engine source for human application [19]. Magnetic propulsion has the advantages of being noninvasive, fuel-free, and harmless to the human body (using a magnetic field below 3 tesla) [27]. In contrast, near-infrared rays have limited penetration depth and are applicable to sites. The acoustic field has the advantage of being used in actual diagnosis despite the limitation of the penetration depth [75].

Most studies are still a proof-of-concept, and their implementation at the cell culture level and in animal models is challenging [101]. Testing the proof-of-concept also focuses on the locomotion of biorobots and not on the evaluation of therapeutic efficacy. Many achievements have been made in the realization of robots that move precisely [102]. However, their sizes are difficult to apply to the human body and their locomotion mechanisms do not consider fluid dynamics [19,103]. Although an increasing number of studies have demonstrated the bioapplicability of biorobots in small animals, this is the only imaging step that tracks their distribution.

Considering the target organs or diseases for which the engine and its fuel work best is a key step in the design of biorobots. It is necessary to carefully examine unmet needs and cooperate with healthcare workers to determine the diseases that require a biorobotic system. Typically, the acidic environment of the stomach is an ideal condition for testing robots, leading to various achievements [14,29,101]. Zhang and Wang evaluated the therapeutic efficacy of nanorobots in an animal model of *Helicobacter pylori* infection [29]. This study demonstrates the feasibility of robotic gastric delivery. However, their advantages over standardized therapies should be considered.

Biorobots were injected into a body to swim at low Reynolds numbers. Reynolds numbers represent the relative magnitude of inertia versus viscous forces, and blood vessels have very low Reynolds numbers. This implies that the existing swimming strategies cannot be applied to biorobot designs. For reference, the Reynolds numbers of the swimmers and microbes differed by 109 [103]. Therefore, many studies have focused on microorganisms or blood cells that swim in bodily fluids [10,34,75,104]. In addition to inspiration from nature, there are other ways to impart momentum while deforming a swimming body to overcome viscous fluid dynamics. However, the problems caused by the friction between cells and proteins that circulate constantly in the body, as well as the Reynolds number, have yet to be studied [105].

Biorobot studies have been limited to identifying movements in the body or in liquid phases [19]. Consequently, few biorobots have been loaded with drugs, and even if they are loaded with drugs, research is often not concerned with the principles of loading and release. Interestingly, there are cases where the mobility of a robot can be applied to a drug release method [36,91]. A plane-shaped biorobot with cardiac microtissue sprayed drugs with an air drop when the engine was turned off. However, as this study was conducted in an ideal cell culture dish environment, considerable consideration should be given to the practical applicability of this system. A novel design was developed to hide sperm inside a 3D-printed magnetic tetrapod and expose the sperm when the four arms of the tetrapod touched cancer cells. Unlike other studies, this approach can be viewed as a more advanced example that considers the therapeutic function at the robot design stage. Finally, the main aim of biorobot development is to develop treatments that go beyond existing therapies. Therefore, further research should focus on the design of drug-delivery methods, specifically loading and release methods.

The biocompatibility of a material is an important issue that must be addressed during the development of biorobots. Living materials such as bacteria can also exclude the possibility of inflammation if they are unintentionally damaged outside the targeted area. For living materials, it is necessary to consider multiple toxicity tests that focus on clinical applicability rather than small animal toxicity test results. In the case of Ni-Ti, a motor that responds to magnetic stimuli, even if biocompatibility is guaranteed owing to the Ti coating, the release of seriously toxic Ni ions should be examined. However, it is difficult to avoid toxicity problems associated with hydrogen peroxide, which is widely used as a fuel for body-deformable engines. On the other hand, DNA materials are very attractive because they are biocompatible and can perform various functions by engineering the nucleotide sequence; however, they always suffer from a short half-life in vivo.

When developing a biorobot, the concept of turning-off features must be designed to avoid operations in unwanted places. Recently, methods for turning off bio-robots after performing a given function have been studied [25]. To turn off the robot, NIR irradiation was used to kill ICG-loaded RBC by triggering hyperthermia-induced cell death [34]. However, this method is limited by the small number of sites available for NIR applications and the insufficient penetration depth. Biorobots using squid-derived proteins have been studied for their ability to release cargo drugs when degraded by low-pH stimulation. This biorobot can be used for intracellular delivery because of its ability to release drugs at a low pH.

In addition to the aforementioned challenges, biorobots face obstacles associated with regulations and manufacturing processes. The components of biorobots are more complicated than those of conventional medical devices. Several issues are associated with large-scale production and validation processes. This may be too early to discuss, but there are many considerations in terms of pharmaceutical regulations, because most materials are rarely used in clinics. The use of microorganisms is an important issue when considering infectivity. However, the living materials that are practically applicable to humans include blood cells. Adopting a method for obtaining and producing patient cells could be a solution to this problem. The conversion of living materials into biomaterials through motif synthesis is safe and offers advantages for process development.

## 7. Conclusions

Recently, significant efforts have been made to establish the feasibility of engines, which are key components of biorobots. Therefore, it is imperative to evaluate the role of the engine in a therapeutic system. Serious consideration is required if there is a demand for biorobots to treat any disease in a clinical setting. The integration of sensing and imaging capabilities is essential for advancing in vivo studies using biorobots. A biorobot should be equipped with a function to detect lesions, and a high-resolution imaging system that can track the biorobot from outside should be developed. Most importantly, current research should focus on drug selection, efficient drug loading, and precise drug release. Overcoming these challenges is expected to attract increased attention for the evolution of miniature surgical solutions.

## Figures and Tables

**Table 1:** Therapeutic cargos delivered by biorobotic delivery systems.

Cargo Type	Therapeutics	Therapeutic Release Mechanism	Robotic DDS Type	Power Source	Targeted Disease/Purpose	Ref
Chemical drug	DOX	Active release by gas generation	Mesoporous silica nanoparticle	Urease	Cancer	[33]
	DOX	pH response	Erythrocytes-based bacterial microswimmer	Bacterial movement + magnetic field	Cancer	[34]
	DOX	Thermal response by NIR	Floating-plane microrobot	Cardiomyocyte contraction	Cancer	[36]
	Fluorouracil	Hyperthermia response	Helical microrobot	Magnetic field	Cancer	[20]
	Clarithromycin	Active release by gas generation	Self-propelled microsphere	Magnesium catalysis	*H. pylori* infection	[29]
	Curcumin, 5-amino salicylic acid	pH response	Self-propelled yeast particle	Glucose oxidase and catalase	Gastric ulcer and colitis	[37]
	Iodine-131	-	Mesoporous silica nanoparticle	Urease	Bladder cancer	[38]
Protein	Caspase 3	pH response	Gold nanowire	Ultrasound	Cancer	[39]
	Staphylococcal α-toxin	pH response	Biomimetic Micromotor	Magnesium catalysis	Oral vaccine for *S. aureus* infection	[40]
	Thrombin	Cargo unlock by aptamer-subtract binding	DNA origami nanorobot	Aptamer conformation	Cancer	[24]
	Cytokine/cell	-	Macrophage-bound iron oxide nanoparticle	Magnetic field	Cancer	[41]
DNA/RNA	Plasmid DNA	Passive release	Artificial bacterial flagella	Magnetic field	Gene disorder diseases	[42]
	siRNA		Gold nanowire	Ultrasound	Gene disorder diseases	[30]
	mRNA	Passive release	Azobenzene lipid nanoparticle	UV/Vis light	Gene transfection	[43]
	Cas9/sgRNA	Passive release	Gold nanowire	Ultrasound	Knock-down of targeted gene	[44]
Cell	MC3T3-E1 fibroblasts, mesenchymal stem cells	Passive release	Magnetic 3D microrobot	Magnetic field	Cell delivery	[45]
	Human adipose–derived mesenchymal stem cell	Passive release	Magnetic 3D microrobot	Magnetic field	Knee cartilage regeneration	[46]
	Olfactory receptor neuron	Pick and drop	Capsule-type microrobot	Magnetic field	Cell delivery	[47]
	CAR-T cell	Passive release	Magnetic beaded modified cells	Magnetic field	Cancer	[48]

## Data Availability

No new data were created or analyzed in this study. Data sharing is not applicable to this study.
